# Are some prepupae and pupae of male mealybugs and root mealybugs (Hemiptera, Coccoidea, Pseudococcidae and Rhizoecidae) mobile?

**DOI:** 10.3897/zookeys.364.6459

**Published:** 2013-12-17

**Authors:** D.J. Williams, Chris J. Hodgson

**Affiliations:** 1 Department of Life Sciences (Entomology), The Natural History Museum, Cromwell Road, London SW7 5BD, U.K.; 2 Department of Biodiversity and Biological Systematics, The National Museum of Wales, Cardiff, Wales, U.K.

**Keywords:** Coccoidea, Pseudococcidae, Rhizoecidae

## Abstract

It is hypothesised here that some mealybug (Pseudococcidae) and root mealybug (Rhizoecidae) prepupae and pupae are mobile. The prepupa and pupa of the mealybug *Promyrmococcus dilli* Williams and the prepupa of the root mealybug *Ripersiella malschae* (Williams) are described and illustrated and their probable mobility is discussed. It is also suggested that the prepupae and pupae of the mealybug *Macrocepicoccus loranthi* Morrison can move rapidly on the leaves when disturbed.

## Introduction and discussion

Most male scale insects (Hemiptera: Coccoidea) only feed in their first and second instars and then pass through non-feeding prepupal and pupal instars before emerging as an adult that can be either wingless or alate. During development of what are now usually termed the archaeococcoid scale insects (Gullan and Cook 2007), the prepupa of alate species may be similar in morphology to the previous feeding stage except that they have no functional mouthparts, and the legs are sometimes reduced in size. Other archaeococcoids that are alate may have a prepupa with developing wing pads and legs. The prepupae and pupae of the morphologically more derived families, the neococcoids, show some signs of legs and the alate species possess developing wing pads. For examples of the life cycles of some families see Danzig (1980) and Gill (1993). Before its moult to a prepupa, the second-instar male usually secretes a covering for the developing prepupa and pupa. It may be a fairly loose covering of wax, an intricate shelter of felted wax, a cocoon-like structure as in the Eriococcidae, or a pupal cell formed of translucent wax as in the Coccidae (Gill 1988). Adult males always emerge backwards from whatever covering protected the prepupal and pupal stages.

**Figure 1.**
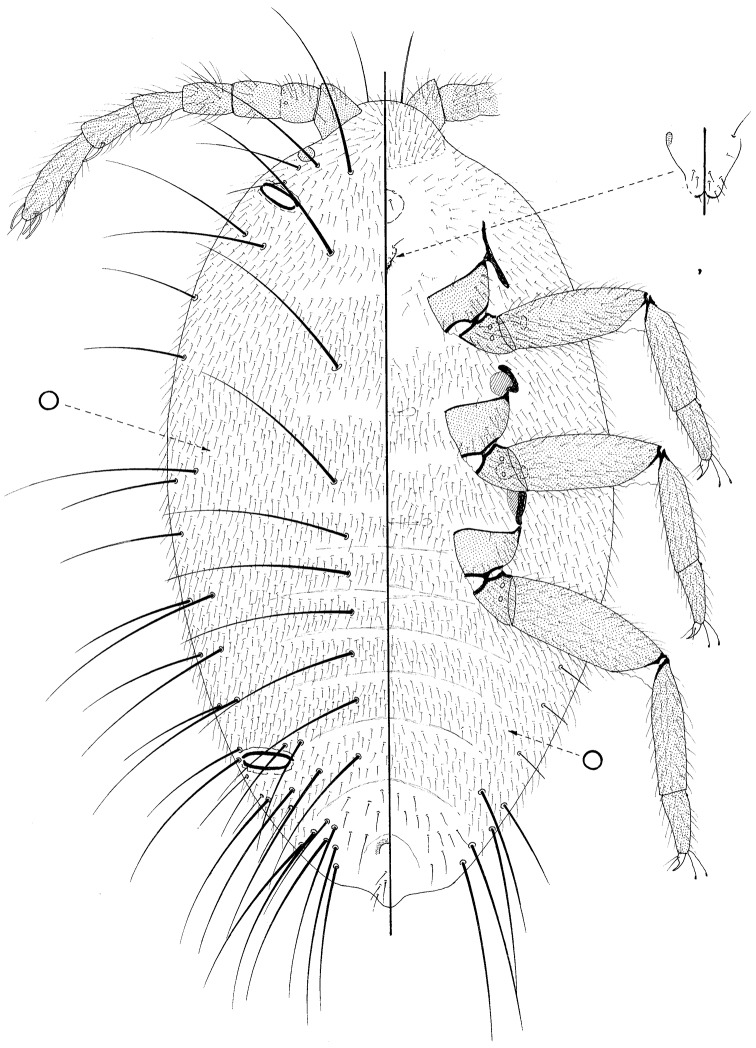
Prepupa of *Promyrmococcus dilli* Williams.

**Figure 2.**
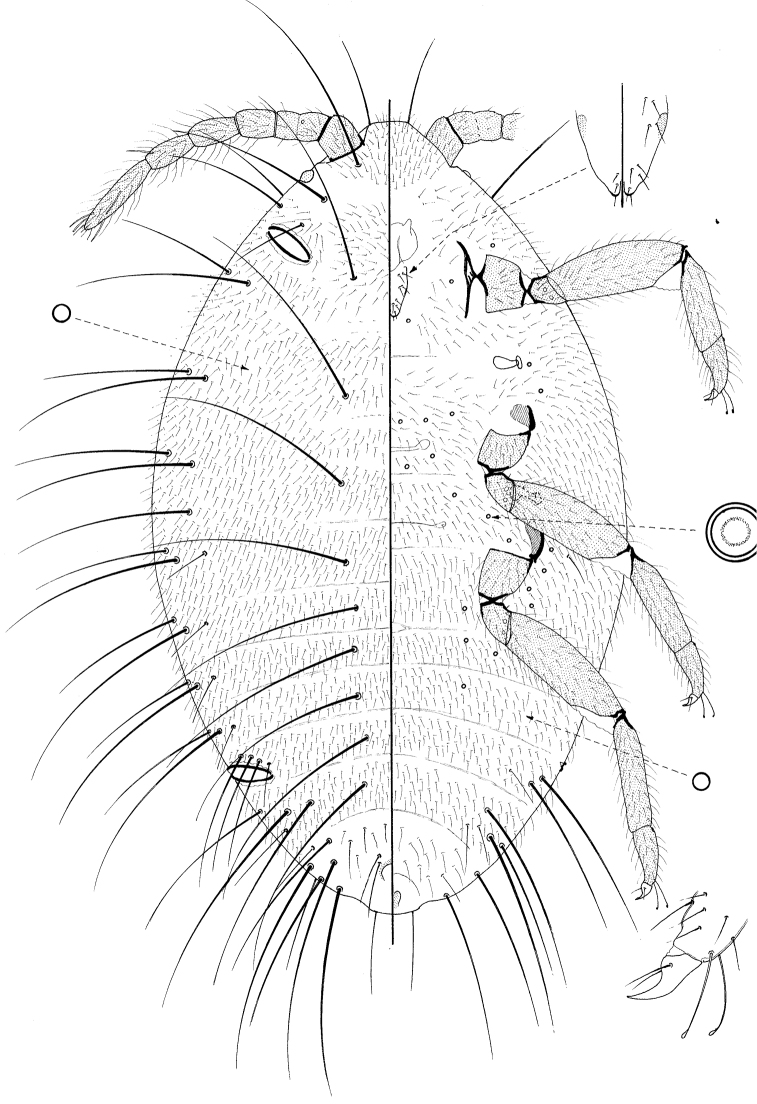
Pupa of *Promyrmococcus dilli* Williams.

**Figure 3.**
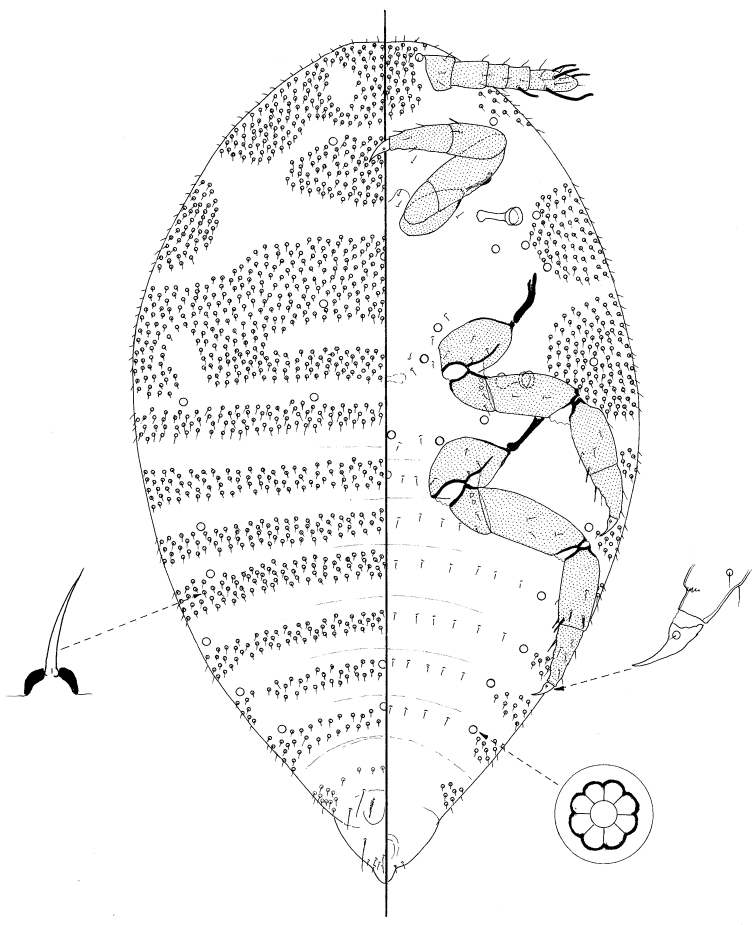
Prepupa of *Ripersiella malschae* (Williams).

Because most male prepupae and pupae develop beneath this waxy cover, the legs are usually non-functional with the claw either showing no signs of development or reduced to a mere point. However, among male mealybugs (Pseudococcidae), there are some taxa in which the prepupa and pupa have relatively well-developed legs, including claws and digitules, that would appear to allow mobility of these insects (Figs 1–3).

Digitules in scale insects are modified setae. The term is usually used for a pair of setae at the base of the claw and a pair at the outer distal end of the tibia of all female instars and the male feeding stages of most taxa. Although sometimes these digitules are setose, many are so-called knobbed or actually spoon-shaped. The adult females of *Steingelia* Nasonov (Steingeliidae) have 12 such digitules on the claw (Morrison 1928), but this is not usual. The claw digitules are often longer than the claw and the tarsal digitules are usually longer than the claw digitules. As far as we know, digitules are present in all first-instar nymphs or crawlers, the main dispersal stage. It has been shown that first-instar nymphs of some armoured scale insects (Diaspididae) attach themselves to their phoretic hosts (Diptera and Coleoptera) by the digitules (Magsig-Castillo et al. 2010). These authors suggest that the swollen ends of each digitule can cling by suction. Regardless of the means by which digitules adhere to surfaces, they must be released rapidly as the nymphs walk. In most illustrations of scale insects that possess legs, the claw is a shown as curved inwards because it is in this position on slide-mounted specimens. In life, however, the claws point outwards so that the plantar surface and the digitules are in contact with a surface.

Digitules reach their fullest development in mealybugs of the tribe Allomyrmococcini (Pseudococcidae) as they are much larger than the claw and are widely expanded distally, sometimes wider than the width of the claw (Williams 1978; Dill et al. 2002). These well-developed digitules are used to climb onto herdsmen ants of the genus *Dolichoderus* when the colony is transported or even onto each other when the ants scoop up a few of the mealybugs when the colony is disturbed. Additional knobbed setae, similar to knobbed digitules, are present also on the lateral surface of the tibiae and tarsi of the adult females of the ant-attended mealybugs *Malaicoccus clavulatus* Williams, *Malaicoccus pilulosus* Williams and *Malaicoccus sumatranus* Williams, and, in addition to these setae, there are knobbed setae on the tibiae and tarsi and body margin in *Thaimyrmococcus daviesi* Williams (Dill et al. 2002; Williams 2004).

Allomyrmococcine mealybugs associated with herdsmen ants are either in the ant trails or in the ants’ nests. It has been shown by Dill et al. (2002) that only about 30% of the mealybugs from an ant-mealybug association is carried in the trail and the remainder are in the nest. Although some gravid females are in the trails, most are present in the ant nests. It was only at the end of the study of the mealybug *Promyrmococcus dilli* Williams by Dill et al. (2002) that adult males, prepupae and pupae were found and these were in nests that had been dropped whole into ethanol. There was no sign of any wax covering on the prepupae or pupae but all stages had well-developed claws and knobbed tarsal digitules (Figs 1, 2). It would be an advantage for prepupae and pupae of mealybugs associated with herdsmen ants to move in the nests and even attach themselves to ants if the nest was moved to another location and the inference is that they are mobile.

Female mealybugs of the genus *Leptococcus* Reyne and *Macrocepicoccus* Morrison have unusually long legs and long slender claws. These mealybugs live on leaf surfaces and are probably parenchyma feeders (Miller and Denno 1977; Williams 2004; Kondo and Gullan 2008). Adult females and nymphs are very active when disturbed. Claws in all of these stages are long and slender. The prepupa and pupa of *Macrocepicoccus loranthi* Morrison and the prepupa of *Leptococcus metroxyli* Reyne possess well-developed tarsal digitules although the claw digitules are short (Miller and Denno 1977). *Macrocepicoccusloranthi* was described from Guyana on *Loranthus* sp. by Morrison (1919) and has been recorded from Colombia (also on *Loranthus* sp.) by Kondo et al. (2008). Recent observations of *Macrocepicoccus loranthi* on *Oryctanthus amplexicatus* (Loranthaceae) show that aggregations of the mealybugs on the leaves are very active when disturbed (Takumasa Kondo, personal communication). This activity is not only confined to the feeding stages but also applies to the prepupae and pupae, which move rapidly. These prepupae and pupae are without any covering or cocoon-like structures and have little wax.

Among the group of root mealybugs based on the genus *Rhizoecus* Künckel d’Herculais, elevated recently to family level, the Rhizoecidae (Hodgson 2012), there are two subfamilies, the Rhizoecinae and the Xenococcinae. Prepupae and pupae of the Xenococcinae have legs without tarsal or claw digitules, and the claws are very poorly developed (Williams 1998). Within the Rhizoecinae, despite the large numbers of species, little is known of their life histories. Mukhopadhyay and Ghose (1996) report that prepupae and pupae of *Rhizoecus amorphophalli* Betrem are without any covering. Adult males of Rhizoecidae are either wingless, brachypterous or alate (Hodgson 2012). Prepupae and pupae of *Ripersiella malschae* (Williams) possess legs with well-developed claws and, although the claw digitules and tarsal digitules are short and setose (Fig. 3), they are similar to those of mobile female stages, as shown in Williams (2004). This species lives in close association with ants of the genus *Pseudolasius* (Malsch et al. 2001; their mealybug #21). The adult male is wingless. It would be an advantage for the prepupae and pupae living in ants’ nests to move and the well-developed claws would help in this process.

It is clear that prepupae and pupae of some Pseudococcidae and probably of the Rhizoecidae can show mobility. Observations on other species of mealybugs and root mealybugs in which the prepupae and pupae have claw and tarsal digitules also may show that these stages can move.

We are taking the opportunity to describe and illustrate the prepupa and pupa of *Promyrmococcus dilli* and the prepupa of *Ripersiella malschae*.

## Pseudococcidae

### Promyrmococcus dilli

Williams

http://species-id.net/wiki/Promyrmococcus_dilli

Promyrmococcus dilli Williams (In Dill et al. 2002: 170).

#### Material studied.

Sabah, Kinabalu, Poring, in nest of *Dolichodorus*, 18.vii.1991, M. Dill (BMNH): 7/2 prepupa (one pharate) + 5 pupae (3 pharate) (good – descriptions taken from non-pharate individuals, with details checked on others).

#### Prepupa

(Fig. 1)

#### Mounted material.

Moderate sized, body 1.23–1.38 mm long, 0.7–0.84 mm widest; oval. Body with numerous very long flagellate setae. Legs and antennae well developed; mouthparts present but lacking stylets; wing buds absent. Ostioles present both anteriorly and posteriorly.

*Dorsum* membranous, segmentation obvious, particularly on abdomen. Each segment densely covered in fine flagellate setae, each 40–50 μm long; also with frequent, extremely long setae with very flagellate apices, each up to about 350 μm long, distributed approximately as follows: with medial pairs on pro-, meso- and metathorax and on abdominal segments I–VI; with 1 long and 1 slightly shorter seta on each side of each segment but with more on abdominal segments VI and VII; with 1–4 slightly shorter setae anterior to each ostiole, and with intermediate fine flagellate setae (each about 100 or so μm long) sparsely throughout. Loculate pores absent but small simple pores frequent throughout, each about 2 μm wide. Ostioles each 90–95 μm wide. Anus about 45 μm wide, with two setae of intermediate length on either side and a pair on posterior body margin.

*Margin* not demarcated; without wing buds. Eyespot 33–35 μm wide.

*Venter* membranous. Circulus present medially between abdominal segments II and III. Fine, flagellate setae similar to those on dorsum, covering most of venter; extremely long flagellate setae only present submarginally on abdominal segments V and VI, and perhaps only marginally on VII; setae of intermediate length infrequent, but present sparsely on VII. Loculate pores, each 6–7 μm wide with an uncertain number of loculi, mainly present medially and submedially on thorax and anterior abdominal segments; simple pores frequent throughout.

Antennae about 515 μm long, 6 segmented but with segment II clearly partially divided and with a campaniform pore present distally on more proximal half of this segment; each segment with many flagellate setae similar to those covering most of body, but with fewest on distal half of segment II; subapical segment with 1 fleshy seta and apical segment with 3 or 4 fleshy setae. Mouthparts clearly present; tentorium barely sclerotized but quite large; labium perhaps 3 segmented, 50–65 μm long, with (on ventral surface) 2 pairs of setae on basal segment, 2 pairs on medial segment and 5 pairs on apical segment; also with 2 pairs on dorsal surface. Spiracles each with peritreme 20–24 μm wide. Legs particularly well developed, lengths (in μm for metathoracic leg): coxa about 120–135; trochanter+ femur 275–310; tibia 190–210; tarsus 95–112; claw 35–38; each trochanter with 2 roundish campaniform pores on each side; each tibia and tarsus without tibial spurs; tarsi one-segmented; tarsi each with a tarsal campaniform pore; tarsal digitules long, extending as long as claw and capitate; claw digitules setose and barely reaching claw apex; claws without a denticle.

#### Pupa

(Fig. 2)

#### Mounted material.

Moderate sized, body 1.20–1.4 mm long, 0.63–0.73 mm widest; oval. Body with numerous very long flagellate setae. Legs and antennae well developed; mouthparts present but very reduced and without stylets; wing buds absent. Ostioles present both anteriorly and posteriorly.

*Dorsum* almost identical to that of the prepupa, and with 0–3 setae of intermediate length on either side of anus.

*Margin* not demarcated, without wing buds. Eyespot 33–35 μm wide.

*Venter* membranous. As for prepupa except loculate pores absent.

Antennae 6 segmented as in prepupa, length about 520–615 μm long. Mouthparts very reduced; tentorium a small, roundish membranous area and labium short, about 35 μm long, with a few setae both dorsally and ventrally. Spiracles each with peritreme 20–28 μm wide. Legs particularly well developed, lengths (in μm for metathoracic leg): coxa about 125–150; trochanter+ femur 295–330; tibia 200–225; tarsus 95–105; claw 35–38; legs otherwise as on prepupa.

#### Comment.

The basic morphology of the prepupa and pupa of *Promyrmococcus dilli* is very similar to that of the adult male (Williams 2004; Hodgson 2012), except that they lack the genital structures, have (at most) 7-segmented antenna (9 segmented on the adult male) and their tarsi appear to be only one segmented. Reduced mouthparts lacking stylets are also present on the adult male.

## Rhizoecidae

### Ripersiella malschae

(Williams)

http://species-id.net/wiki/Ripersiella_malschae

Rhizoecus malschae Williams, 2004: 779.
Ripersiella malschae (Williams): Kozár and Konczné Benedicty 2007: 495.

#### Prepupa

(Fig. 3)

#### Material studied.

Paratype, Sabah, Kinabalu Park, Poring Hot Springs, with *Pseudolasius*, 28.iii.1998, A. Malsch (BMNH): 1/1 pharate prepupa (good, but distribution of pores and leg setae difficult to ascertain as pupa fairly-well developed).

#### Mounted material.

Small, body 508 μm long, 286 μm widest, oval but rather pointed posteriorly. Legs and antennae well developed; mouthparts and wing buds absent.

*Dorsum* membranous, segmentation obvious, particularly on abdomen. Each segment with a dense band of short setae, each 5–7 μm long on a convex basal socket; bands narrowest on posterior segments. With 3 pairs of long setae on posterior-most segments, each 23–30 μm long; incipient penial sheath with a group of about 18 setae, similar to those on rest of dorsum. With loculate pores, each about 6 μm wide with mainly 8 loculi, near margins of abdominal segments II–VI and also on metathorax.

*Margin* not demarcated; without wing buds.

*Venter* membranous. Small setae, similar to those covering most of dorsum, present anteriorly and laterally on head, in large broad groups laterally on pro- and mesothorax, and in small groups laterally on metathorax and abdominal segments II–VII; somewhat similar setae also present very sparsely medially across all segments except perhaps prothorax. Loculate pores similar to those on dorsum present submarginally on abdominal segments and sparsely medially on all thoracic segments and head.

Antennae 6 segmented, about 100 μm long; pedicel very short, about 10 μm long; all segments with a few setose setae; subapical segment with a fleshy seta and apical segment with 3 or 4 fleshy setae. Mouthparts absent. Spiracles each with peritreme 16–18 μm wide. Legs particularly well developed, lengths (metathoracic leg in μm): coxa about 50; trochanter + femur 88; tibia + tarsus 85; claw 21; each trochanter with 2 roundish campaniform pores on each side; each tibia with 2 tibial spurs on distal ventral margin but also with perhaps 2 more laterally; tarsi with a spur-like seta on ventral margin near proximal end; tarsal campaniform pores present but tarsal digitules considered to be absent; claw digitules present but minute; claws without a denticle. Anus apparently on ventral surface.

#### Comment.

The prepupa of *Ripersiella malschae* looks similar to the adult male (Hodgson 2012) but lacks the well-developed penial sheath. In addition, the loculate pores on the prepupa clearly have mainly 8 loculi whereas they appeared to have 5 or fewer on the adult male.

## Supplementary Material

XML Treatment for Promyrmococcus dilli

XML Treatment for Ripersiella malschae
